# Iron Accumulation and Lipid Peroxidation in Cellular Models of Nemaline Myopathies

**DOI:** 10.3390/ijms26041434

**Published:** 2025-02-08

**Authors:** Alejandra López-Cabrera, Rocío Piñero-Pérez, Mónica Álvarez-Córdoba, Paula Cilleros-Holgado, David Gómez-Fernández, Diana Reche-López, Ana Romero-González, José Manuel Romero-Domínguez, Mario de la Mata, Rocío M. de Pablos, Susana González-Granero, José Manuel García-Verdugo, José A. Sánchez-Alcázar

**Affiliations:** 1 Centro Andaluz de Biología del Desarrollo (CABD-CSIC-Universidad Pablo de Olavide), 41013 Sevilla, Spain; alopcab2@alu.upo.es (A.L.-C.); rpieper@alu.upo.es (R.P.-P.); malvcor@upo.es (M.Á.-C.); pcilhol@upo.es (P.C.-H.); dgomfer1@acu.upo.es (D.G.-F.); dreclop@alu.upo.es (D.R.-L.); aromgon1@upo.es (A.R.-G.); jmromdom@upo.es (J.M.R.-D.); 2 Departamento de Fisiología, Facultad de Ciencias de la Salud, Universidad de Granada, 51001 Ceuta, Spain; mrdelamata@ugr.es; 3 Departamento de Bioquímica y Biología Molecular, Facultad de Farmacia, Universidad de Sevilla, 41012 Sevilla, Spain; depablos@us.es; 4 Instituto de Biomedicina de Sevilla (IBiS), Hospital Universitario Virgen del Rocío (HUVR)-CSIC-Universidad de Sevilla, 41013 Sevilla, Spain; 5 Laboratory of Comparative Neurobiology, Cavanilles Institute of Biodiversity and Evolutionary Biology, University of Valencia and CIBERNED-ISCIII, 46980 Valencia, Spain; susana.gonzalez@uv.es (S.G.-G.); j.manuel.garcia@uv.es (J.M.G.-V.)

**Keywords:** nemaline myopathy, iron accumulation, lipid peroxidation

## Abstract

One of the most prevalent types of congenital myopathy is nemaline myopathy (NM), which is recognized by histopathological examination of muscle fibers for the presence of “nemaline bodies” (rods). Mutations in the actin alpha 1 (*ACTA1*) and nebulin (*NEB*) genes result in the most prevalent types of NM. Muscle weakness and hypotonia are the main clinical characteristics of this disease. Unfortunately, the pathogenetic mechanisms are still unknown, and there is no cure. In previous work, we showed that actin filament polymerization defects in patient-derived fibroblasts were associated with mitochondrial dysfunction. In this manuscript, we examined the pathophysiological consequences of mitochondrial dysfunction in patient-derived fibroblasts. We analyzed iron and lipofuscin accumulation and lipid peroxidation both at the cellular and mitochondrial level. We found that fibroblasts derived from patients harboring *ACTA1* and *NEB* mutations showed intracellular iron and lipofuscin accumulation, increased lipid peroxidation, and altered expression levels of proteins involved in iron metabolism. Furthermore, we showed that actin polymerization inhibition in control cells recapitulates the main pathological alterations of mutant nemaline cells. Our results indicate that mitochondrial dysfunction is associated with iron metabolism dysregulation, leading to iron/lipofuscin accumulation and increased lipid peroxidation.

## 1. Introduction

Congenital myopathy is a genetically heterogeneous group of hereditary muscle diseases categorized according to the histological characteristics identified in muscle biopsy [1,2]. Specifically, nemaline myopathy (NM) is the most common subtype of congenital myopathy, characterized by the presence of inclusions in muscle fibers known as nemaline bodies. NM, a rare genetic skeletal muscle disease affecting 1 in 50,000 live births [3,4], was first described in 1963 by Conen et al. [5] and Shy et al. [6]. NM manifests clinical features such as muscle weakness and hypotonia, decreased respiratory function, and impaired motor development.

Currently, 14 genes have been identified as responsible for NM [2]. Most of the genes code for components of the thin filament of the sarcomere or additional structural or regulatory components to the sarcomere. The genes most associated with NM are *ACTA1* and *NEB* genes, which code for actin alpha 1 (ACTA1) and nebulin (NEB) proteins, respectively [7]. It is estimated that over 50% of NM cases are caused by mutations in the *NEB* gene, while 15–20% can be attributed to mutations in *ACTA1* gene. Due to its large molecular weight (600–800 kDa), NEB is often referred to as the giant actin-binding protein. It is crucial for maintaining and controlling the length of the actin filament. Actin isoform ACTA1 is predominantly located in the thin filaments of skeletal muscles and is necessary for muscular contraction together with myosin [2,8].

The diagnosis of NM is mainly made by examining histopathological findings in muscle biopsies [9]. Histopathological alterations in NM are characterized by the presence of nemaline bodies, which are electron-dense protein aggregates that are distributed in the form of rods, which can be visualized within the muscle fibers of muscle biopsies from patients [9,10,11]. Disorganized sarcomere Z-disk proteins, including actin, tropomyosin, myotilin, γ-phylamine, cofilin-2, tetonin, nebulin, and actin-like thin filaments, constitute the protein aggregates of these nemaline bodies [7,8]. In addition, muscle enzyme levels such as creatinine kinase are analyzed, which may show normal or slightly elevated values. However, the origin of these nemaline bodies is uncertain, although, because of the accumulated proteins, they are believed to be derived from the Z-line of the sarcomere [12].

Consequently, histopathological findings obtained with electron microscopy are the main evidence for the diagnosis of the disease. In addition to the nemaline bodies, other peculiarities can be found in muscle biopsies, such as changes in the proportion and size of muscle fibers, since patients with NM present a predominance of type I (slow twitch) muscle fibers [7,10].

NM disease has been related to mitochondria dysfunction, particularly complex I dysfunction or deficiency [13,14]. It has been described that *ACTA1* mutants present a “down regulation” of mitochondrial complex I due to the disruption of the integrity of the thin filaments, impaired muscle function, and a reduced production of adenosine triphosphate (ATP) [14]. Furthermore, actin filaments are crucial for the correct mitochondrial morphology and function [15,16,17]. Therefore, actin filaments mainly control mitochondrial dynamics, metabolism, trafficking, and autophagy [18,19,20,21].

The close connections between mitochondria dysfunction and actin cytoskeleton—including actin filament depolymerization, a deficiency of mitochondrial bioenergetics, and a downregulation of the expression levels of mitochondrial proteins—were described in a previous investigation by our group [16].

Elevated intracellular iron levels are associated with a wide range of pathological conditions [22,23]. Iron is an essential element for several cellular processes, serving as a cofactor for enzymes involved in oxygen metabolism (such as oxidases, peroxidases, catalases, and hydroxylases), electron transport (cytochromes), and oxygen delivery (hemoglobin). It also plays a vital role in neuronal function and the immune system [24]. Within cells, iron is primarily utilized in the mitochondria, where it contributes to the biosynthesis of heme and iron–sulfur clusters [22]. Iron overload can participate in the generation of reactive oxygen species (ROS) through Fenton reaction, causing oxidative stress, decreasing respiratory function, and damaging mitochondrial DNA and cellular components such as lipids [25,26,27].

The relationship between mitochondrial dysfunction and iron accumulation was recently examined by our research group in several rare neurogenetic disorders under the classification of neurodegeneration with brain iron accumulation (NBIA), such as β-propeller protein-associated neurodegeneration (BPAN) [25] and pantothenate kinase-associated neurodegeneration (PKAN) [28]. Moreover, this connection has been also demonstrated in rare mitochondrial diseases induced by mutation in lipoyltransferase 1 (LIPT1) gene [29].

This study aimed to analyze intracellular iron content and lipid peroxidation in fibroblasts derived from two patients with *ACTA1* and two patients with *NEB* pathogenic variants. Additionally, we examined the effect of actin depolymerizing agents such as ROCK (Rho-associated protein kinase) inhibitors and Cytochalasin D on control fibroblasts to confirm whether actin depolymerization mimics the physiopathology of NM patient-derived fibroblasts by analyzing intracellular iron content, lipid peroxidation, and the expression levels of proteins involved in iron metabolism, among others.

## 2. Results

### 2.1. NM Patient-Derived Fibroblasts Show Iron Accumulation

**Figure 1:**
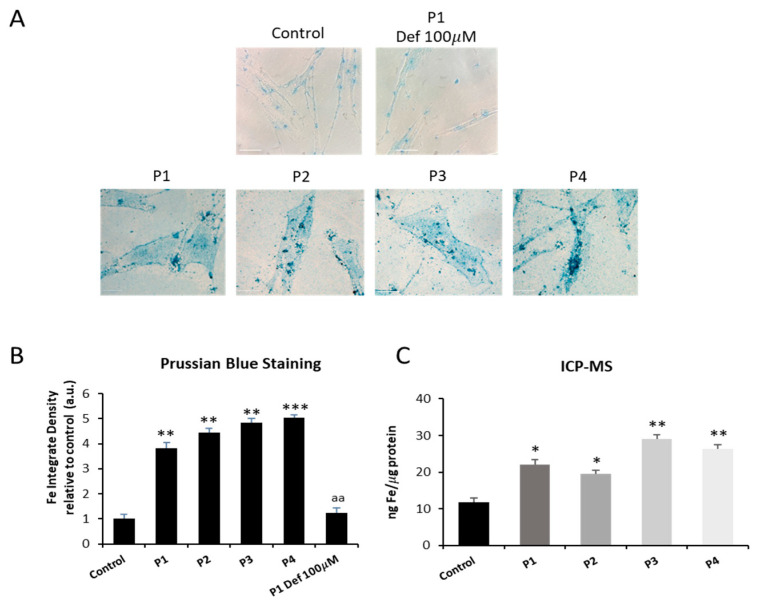
Iron accumulation in NM cells. (**A**) Prussian Blue staining of control cells (C1) and NM fibroblasts (P1, P2, P3, and P4) was carried out as described in Section 4, Materials and Methods. P1 fibroblasts were exposed to 100 µM deferiprone (Def), an iron chelating agent, for 24 h as a negative control. Images were made in brightfield by an Axio Vert A1 inverted optical microscope (Zeiss, Oberkochen, Germany) with a 40× objective and were analyzed using Fiji-ImageJ software (version 2.9.0/1.53t) (National Institute of Health, Bethesda, MD, USA). Scale bar = 20 µm. (**B**) Quantification of Prussian Blue staining images was performed by the Image J software (version 1.54f). (**C**) Iron content determined by ICP-MS in NM fibroblast. ICP-MS was used to measure the total iron content of control and NM patients as detailed in the Material and Methods. Data represent the mean ± SD of three separate experiments. * *p* < 0.05, ** *p* < 0.01, *** *p* < 0.001 between NM fibroblasts and controls; ^aa^ *p* < 0.01 between untreated and treated NM cells between the presence and the absence of deferiprone (Def). A.U., arbitrary units.

As mitochondrial dysfunction can be associated with impaired iron metabolism [30], we first examined intracellular iron accumulation by Prussian Blue staining in both control and NM fibroblasts P1, P2, P3, and P4. The results revealed a significant increase in iron staining in NM mutant cells compared to controls. To serve as a negative control and validate the specificity of Prussian Blue staining for iron, P1 fibroblasts were treated with deferiprone (Def), an iron-chelating drug (Figure 1A,B and Figure S1). To confirm the abnormal cellular iron content in NM fibroblasts, we next determined intracellular iron levels by inductively coupled mass spectrometry (ICP-MS). Mutant NM fibroblasts P1, P2, P3, and P4 showed a significant increase in total iron content with respect to control cells (Figure 1C).

### 2.2. NM Patient-Derived Fibroblasts Present Lipofuscin-like Aggregate Accumulation

**Figure 2:**
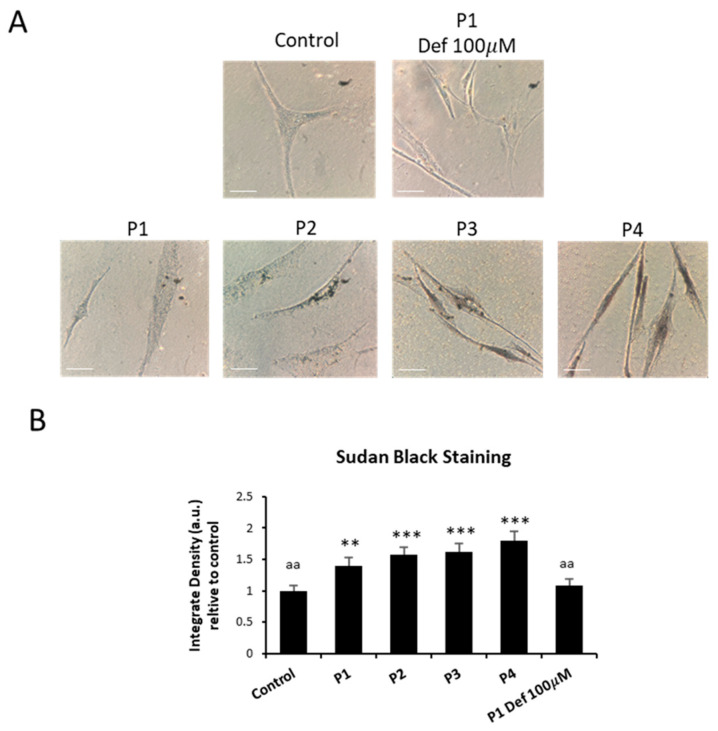
Lipofuscin accumulation in NM fibroblasts. (**A**) Sudan black staining of control cells (C1) and NM fibroblasts (P1, P2, P3, and P4) was performed as described in Section 4, Materials and Methods. P1 fibroblasts were exposed to 100 µM deferiprone (Def), an iron chelating agent, for 24 h, as a negative control. Images were made in brightfield by an Axio Vert A1 inverted optical microscope (Zeiss, Oberkochen, Germany) with a 40× objective and were analyzed using Fiji-ImageJ software (version 2.9.0/1.53t) (National Institute of Health, Bethesda, MD, USA). Scale bar = 20 µm. (**B**) Quantification of Sudan Black in control and NM fibroblasts was performed by ImageJ software (version 1.54f). Data represent the mean ± SD of three separate experiments. ** *p* < 0.01, *** *p* < 0.001 between NM fibroblasts and controls; ^aa^ *p* < 0.01 between untreated and treated NM cells between the presence and the absence of deferiprone (Def). A.U., arbitrary units.

**Figure 3:**
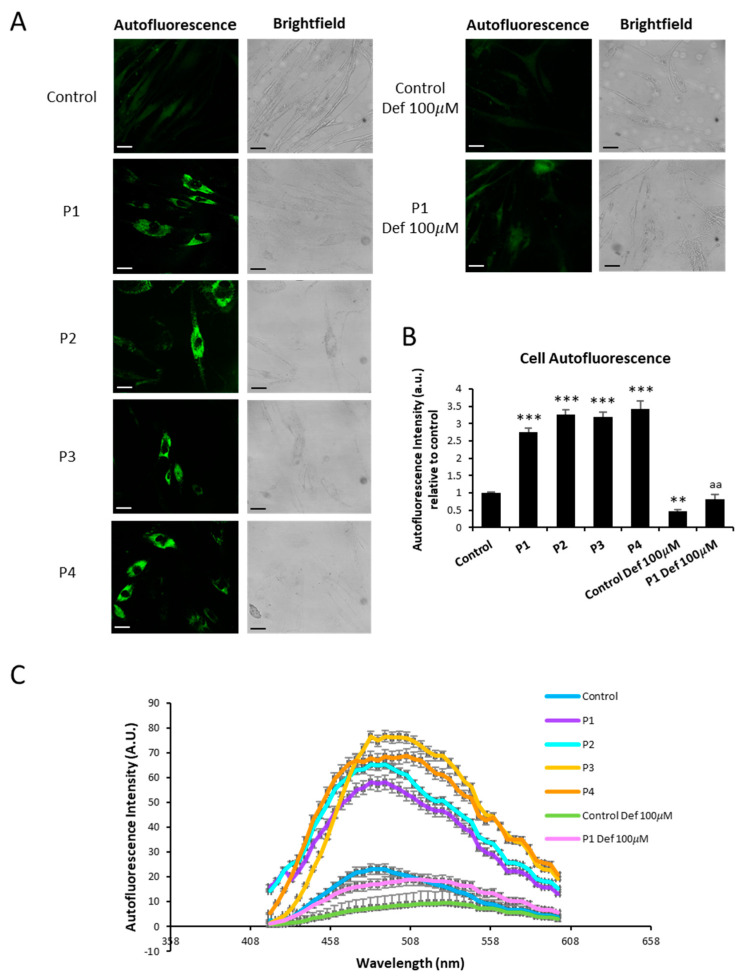
Lipofuscin-like aggregates in NM fibroblasts. (**A**) Representative autofluorescence and bright field (BF) images of control (C1) and NM fibroblasts (P1, P2, P3, and P4). Control cell and P1 were treated with 100 μM deferiprone (Def) for 24 h as a negative control. Scale bar = 20 μm. (**B**) Quantification of cell autofluorescence was determined by image analysis using the Fiji software (version 2.9.0/1.53t). (**C**) The autofluorescence spectra of lipofuscin granules were measured by confocal laser scanning microscopy (Nikon A1R, Shinagawa, Tokyo, Japan) in control, NM fibroblasts (P1, P2, P3, and P4), and negative controls (control and P1 treated with 100 μM deferiprone (Def)). Excitation laser source: 405 nm. The emission spectra were recorded in 20 large lipofuscin granules in 20 cells. Results are expressed as mean ± SD of autofluorescence intensity. ** *p* < 0.01, *** *p* < 0.001 between NM fibroblasts and controls; ^aa^ *p* < 0.01 between the presence and the absence of DEF in controls and Patient 1 cells. A.U., arbitrary units.

As abnormal accumulation of lipofuscin has been reported in several cell types presenting iron overload [28,31] and lipofuscin accumulation can result from lipid peroxidation, a process stimulated by iron [24], we next examined the presence of lipofuscin by Sudan Black staining and autofluorescence analysis in control and NM fibroblasts. Compared to control cells, NM mutant cells displayed significantly higher levels of Sudan Black staining (Figure 2A,B and Figure S2). In addition, NM fibroblasts presented higher levels of autofluorescence (Figure 3A,B), indicating increased lipofuscin accumulation.

Autofluorescence and Sudan Black staining in patient fibroblasts were significantly reduced after treatment with 100 µM deferiprone (Def) [28], suggesting that iron is contributing to the accumulation of the autofluorescence and Sudan Black-positive lipofuscin-like material (Figure 2A,B, Figure 3A,B and Figure S2). Furthermore, to confirm the lipofuscin-like features of the aggregates, the fluorescence spectral characteristics of lipofuscin granules in NM cells were measured by confocal laser scanning microscopy. Under excitation at 405 nm, lipofuscin granules showed an emission peak at 520–540 nm (Figure 3C). These results are consistent with the characteristics of lipofuscin granules observed in retinal pigment epithelial cells [32] and in pantothenate kinase-associated neurodegeneration (PKAN) cellular models [28].

**Figure 4:**
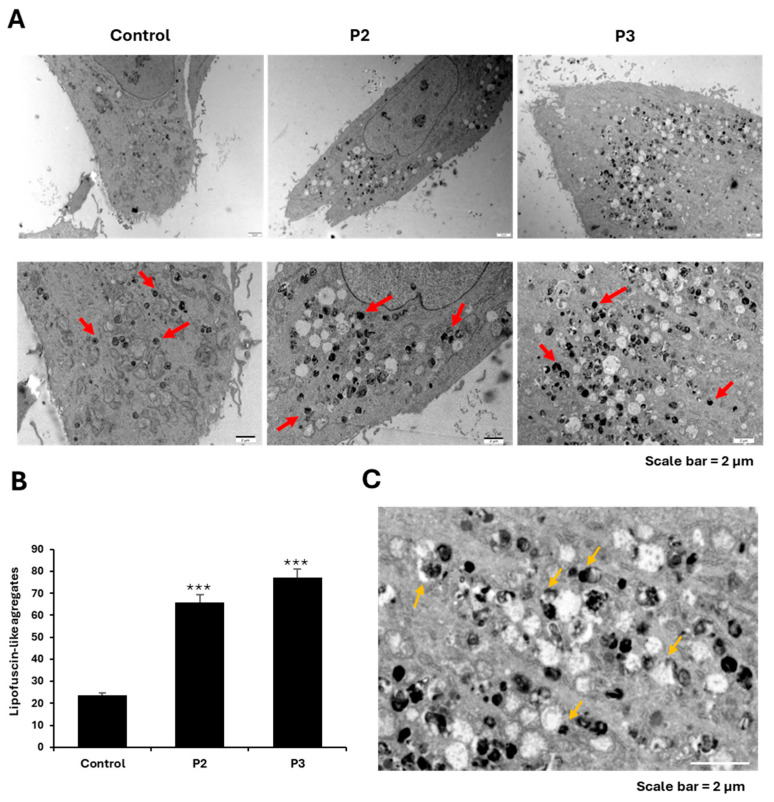
Electron microscopy images of control and P2 (*ACTA1*) and P3 (*NEB*) fibroblasts. (**A**) Representative electron microscopy images of control (C1) and NM fibroblasts (P2 and P3). Red arrows were used to highlight the lipofuscin-like granules. (**B**) Quantification of lipofuscin-like aggregates. (**C**) P2 cells showed mitochondrial vacuolization, and condensation/lateralization of mitochondrial membranes (orange arrow). Scale bar = 2 μm. Data represent the mean ± SD of the examination of 50 cells per condition. *** *p* < 0.001 between NM cells and controls.

The presence of elevated intracellular lipofuscin-like granules in NM fibroblasts was validated using TEM analysis (Figure 4A,B). In addition, the examination of mitochondrial alterations in NM cells showed mitochondrial vacuolization and condensation of damaged areas of mitochondrial membranes, which eventually were extruded to the cytosol, forming dense lipofuscin-like granules (Figure 4C).

### 2.3. Mitochondrial Iron Accumulation in NM Fibroblasts

**Figure 5:**
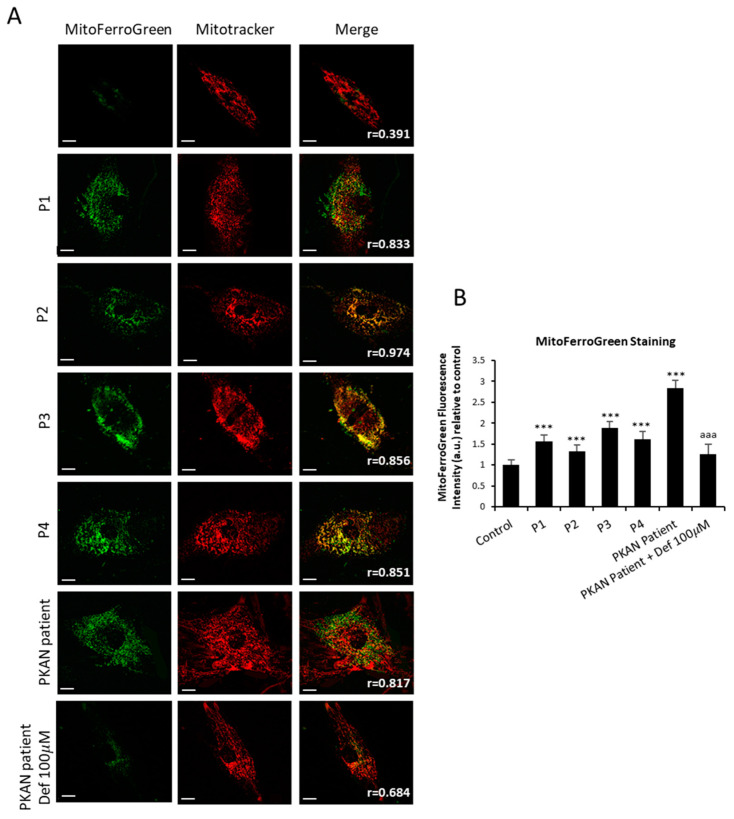
Mitochondrial ferrous iron (Fe^2+^) in control and NM cells. (**A**) Levels of mitochondrial ferrous iron in control (C1) and NM fibroblasts (P1, P2, P3, and P4) were assayed by MitoFerroGreen staining as described in Materials and Methods. Cellular models of pantothenate kinase-associated neurodegeneration (PKAN) were used as a positive control. PKAN cells were exposed to 100 µM deferiprone (Def), an iron chelating agent, for 24 h as a negative control. (**B**) MitoFerroGreen staining quantification was conducted by using Fiji software (version 2.9.0/1.53t). Cells were incubated with MitoTracker^TM^ Deep Red FM to demonstrate that MitoFerroGreen signal colocalizes with a mitochondrial marker. The colocalization of both markers was assessed by the DeltaVision software (version softWoRx 7.0; Applied Precision; Issaquah, Washington (WA), United states (USA)) calculating the Pearson correlation coefficient. Pearson correlation coefficient was >0.75 in NM fibroblasts and control. Scale bar = 20 µm. *** *p* < 0.001 between control and NM fibroblasts; ^aaa^ *p* < 0.001 between the presence and the absence of DEF between treated and untreated PKAN patient cells. Data represent the mean ± SD of four separate experiments. A.U., arbitrary units.

Next, we examined the levels of mitochondrial Fe^2+^ by MitoFerroGreen staining. This assay was carried out in control, mutants’ fibroblasts P1 and P2 harboring *ACTA1* mutations, and P3 and P4 harboring *NEB* mutations (Figure 5A). Mutant NM cells presented elevated levels of mitochondrial Fe^2+^. Cellular models of pantothenate kinase-associated neurodegeneration (PKAN) were used as a positive control [28]. Additionally, PKAN-derived fibroblasts were treated with 100 µM deferiprone as a negative control. Colocalization analysis with MitoTracker^TM^ Deep Red FM, a mitochondria marker, demonstrated that the signal colocalizes with MitoFerroGreen staining (Pearson correlation coefficient > 0.75).

### 2.4. Lipid Peroxidation in NM Fibroblasts

**Figure 6:**
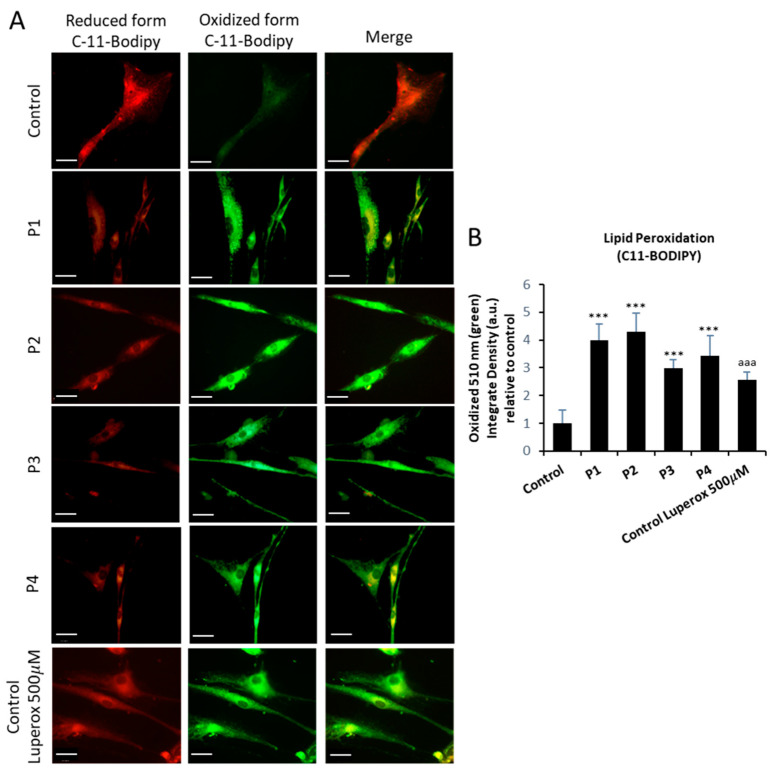
Cellular lipid peroxidation in control and NM cells. (**A**) Levels of cellular lipid peroxidation in control (C1) and NM fibroblasts (P1, P2, P3, and P4) were measured using BODIPY^®^ staining as detailed in Section 4, Material and Methods. (**B**) BODIPY^®^ staining quantification was performed by using the Fiji software (version 2.9.0/1.53t). Images were made in by an Axio Vert A1 inverted optical microscope (Zeiss, Oberkochen, Germany) with a 40× objective. Control fibroblasts were treated with 500 µM Luperox (Tert-butyl hydroperoxide) for 15 min as positive control of lipid peroxidation. Scale bar = 20 µm. *** *p* < 0.001 between control and NM fibroblasts; ^aaa^ *p* < 0.001 between the presence and the absence of Luperox between treated and untreated Control cells. Data represent the mean ± SD of four separate experiments. A.U., arbitrary units.

**Figure 7:**
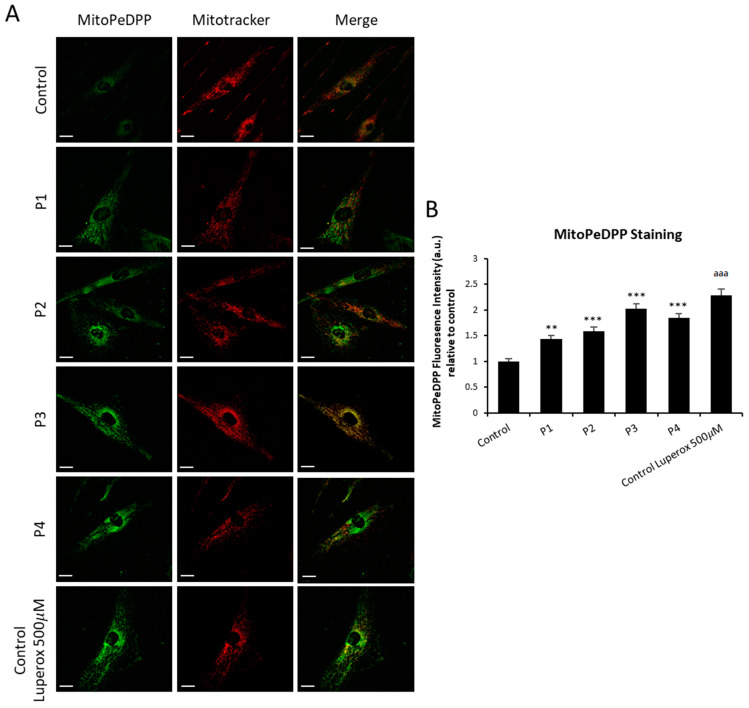
Mitochondria lipid peroxidation in control and NM cells. (**A**) Levels of mitochondrial lipid peroxidation in control (C1) and NM fibroblasts (P1, P2, P3, and P4) were assayed by MitoPeDPP staining as detailed in Section 4, Material and Methods. (**B**) MitoPeDPP staining quantification was performed by using the Fiji software (version 2.9.0/1.53t). Cells were incubated with MitoTracker^TM^ Deep Red FM to demonstrate that MitoPeDPP signal colocalizes with a mitochondrial marker. Control fibroblasts were treated with 500 µM Luperox (Tert-butyl hydroperoxide) for 15 min as positive control of lipid peroxidation. Colocalization of both markers MitoPeDPP and MitoTracker^TM^ was assessed by the DeltaVision software (version softWoRx 7.0Applied Precision; Issaquah, Washington (WA), United states (USA)). Scale bar = 20 µm. ** *p* < 0.01, *** *p* < 0.001 between control and NM fibroblasts; ^aaa^ *p* < 0.001 between the presence and the absence of Luperox between treated and untreated control cells. Data represent the mean ± SD of four separate experiments. A.U., arbitrary units.

As iron accumulation in NM cells can induce the oxidation of intracellular lipids by Fenton reaction [24], we next addressed cellular and mitochondrial lipid peroxidation by BODIPY^®^ and MITOPeDPP staining, respectively. Cellular lipid peroxidation was notably increased in mutant NM cells (Figure 6A,B). In addition, mutant NM cells showed increased levels of mitochondrial lipid peroxidation (Figure 7A,B). Control fibroblasts were treated with 500 µM Luperox (Tert-butyl hydroperoxide) as a positive control of lipid peroxidation.

### 2.5. NM Patient-Derived Fibroblasts Also Presented Altered Expression Levels of Proteins Involved in Iron Metabolism

**Figure 8:**
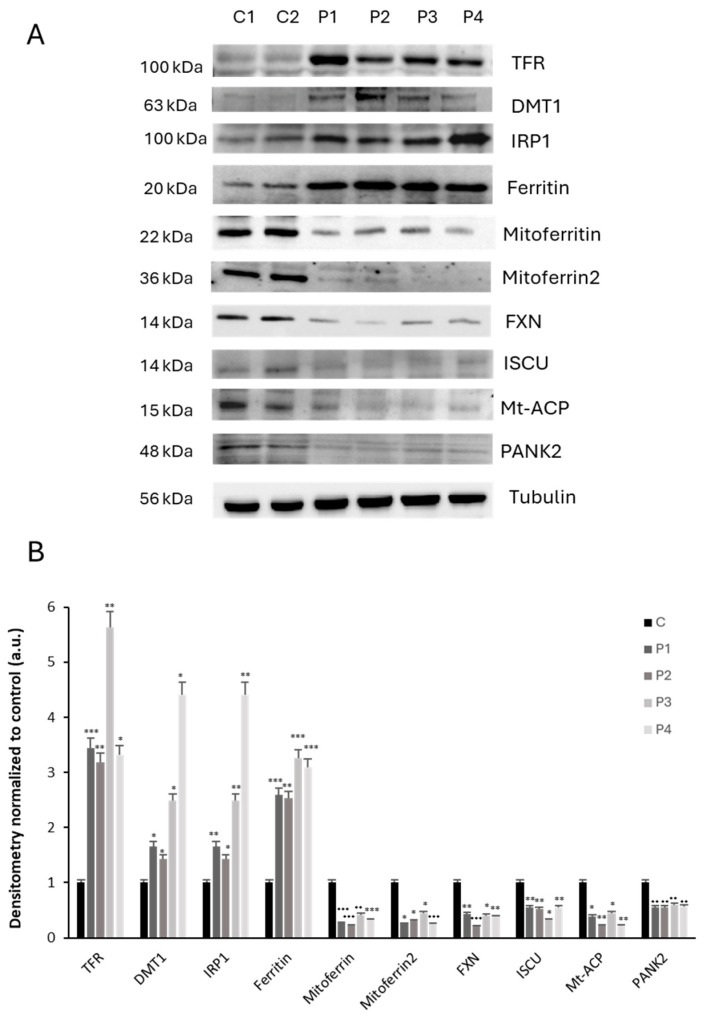
Iron metabolism-related protein expression levels in NM fibroblast. (**A**) Immunoblotting analysis of cellular extracts from controls (C1 and C2) and NM patient cell lines P1, P2, P3, and P4. Protein extracts (50 μg) were separated on a SDS polyacrylamide gel and immunostained with antibodies against TFR, DMT1, IRP1, Ferritin, Mitoferritin, Mitoferrin2, FXN, ISCU, Mt-ACP, and PANK2. Tubulin was used as a loading control. (**B**) Densitometry of Western blotting. For control cells (C1 and C2), data are the mean ± SD of the two control cell lines. Data represent the mean ± SD of three separate experiments. * *p* < 0.05, ** *p* < 0.01, *** *p* < 0.001 between NM cells and controls. A.U., arbitrary units.

We next assessed iron metabolism dysregulation by examining the expression levels of key proteins involved in iron trafficking, storage, and regulation, such as TFR1, DMT1, IRP1, Ferritin, Mitoferritin, Mitoferrin2, FXN, ISCU, Mt-ACP, and PANK2. NM mutant fibroblasts showed increased expression levels of TFR1, DMT1, IRP1, and Ferritin respect to control cells (Figure 8A). As impaired iron metabolism has been previously associated with defective ISC (iron-sulfur cluster) biogenesis [33,34], we also explored the expression levels of FXN and ISCU in control and NM fibroblasts. Expression levels of FXN and ISCU were dramatically reduced in NM fibroblasts, suggesting disorganization of the ISC complex (Figure 8A). 

Moreover, we examined the expression level of IRP1 involved in the control of iron metabolism. We observed a significant increase of IRP1, suggesting a misregulation in iron metabolism.

**Figure 9:**
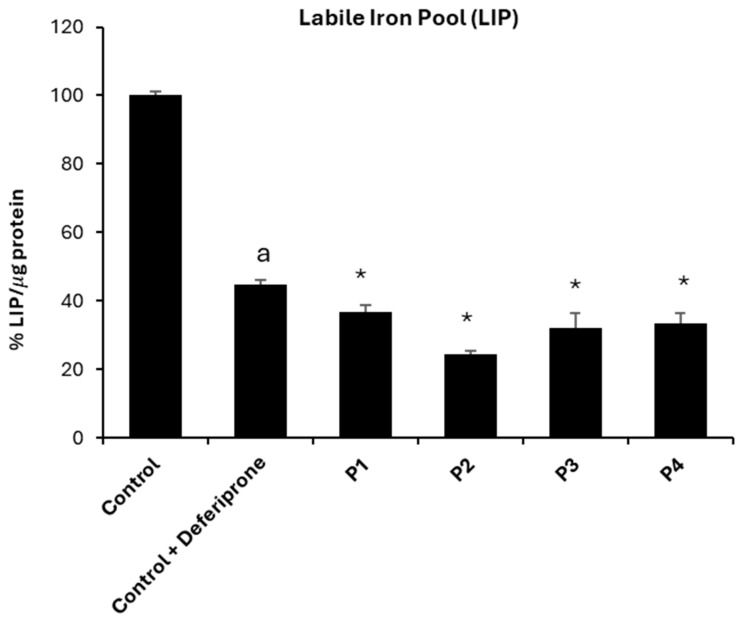
Labile iron pool (LIP). LIP in control (C1) and NM fibroblasts (P1, P2, P3, and P4) were measured as described in Section 4, Materials and Methods. As a negative control, control cells were exposed to 100 μM deferiprone (Def) for 24 h. For control cells, data are mean ± SD of three control cell lines. * *p* < 0.05 between control and NM fibroblasts. ^a^ *p* < 0.05 between the presence and the absence of DEF between treated and untreated Control cells. Data represent the mean ± SD of six separate experiments.

In addition, the levels of labile iron pool (LIP) were also quantified by a calcein assay. Interestingly, LIP was significantly decreased in NM cells, indicating a deficiency of free iron and abnormal iron handling by mutant cells (Figure 9).

### 2.6. Actin Polymerization Defects and Mitochondrial Dysfunction Are Present in Control Fibroblasts Treated with Actin Depolymerizing Agents

**Figure 10:**
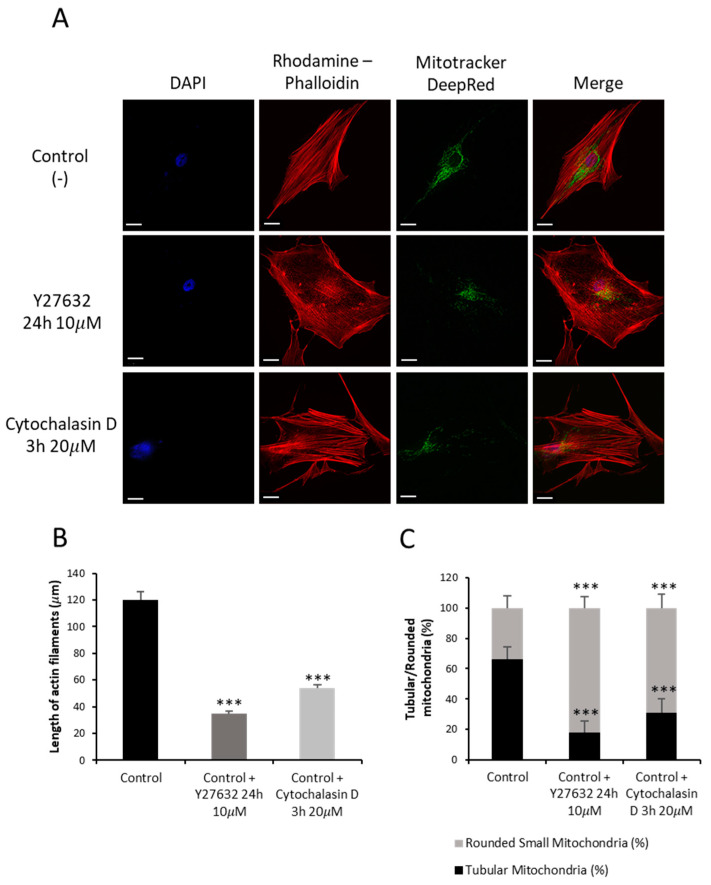
Actin staining by Rhodamine–Phalloidin and Mitotracker^TM^ DeepRed of control fibroblasts treated with Y27632 or Cytochalasin D inhibitors. (**A**) Control fibroblasts (C1) were treated with 10 µM Y27632 for 3 h or with 20 µM Cytochalasin D for 24 h. Control fibroblasts were stained with Rhodamine–Phalloidin and Mitotracker^TM^ DeepRed and visualized under a widefield fluorescence microscope. Nuclei were revealed by DAPI staining. Treated control fibroblasts presented smaller and unstructured actin filaments compared to untreated control fibroblasts. Images were taken using the 40× lens and processed by the ImageJ software (version 1.54f). (**B**) Measurement of the length of actin filaments (µm). The length of the actin filaments was measured in triplicate with the ImageJ software (version 1.54f) in 30 images. (**C**) Quantification of tubular and rounded percentage of mitochondria in control cells. Data represent the mean ± SD of three separate experiments (at least 100 cells for each condition and experiment were analyzed). *** *p* < 0.001 between treated and untreated control cells. Scale bar = 20 µm.

Since in our previous work we demonstrated that actin polymerization defects were associated with mitochondrial dysfunction in NM-derived fibroblasts [16], we hypothesized that the treatment of control fibroblasts with actin depolymerizing agents such as Y27632 (ROCK inhibitor) or Cytochalasin D would mimic the NM fibroblast physiopathology. Rhodamine–Phalloidin staining of control cells treated with Y27632 or Cytochalasin D showed unstructured actin filaments of shorter length compared to untreated control fibroblasts (Figure 10A,B). Furthermore, control cells were stained with Mitotracker^TM^ DeepRed, a mitochondrial marker, to analyze the mitochondrial morphology by fluorescence microscopy. Comparing treated and untreated control fibroblasts, we observed that treated fibroblasts with both inhibitors presented altered mitochondrial morphology along with a lower percentage of tubular mitochondria (Figure 10C).

### 2.7. Alterations in Mitochondrial Bioenergetics Are Presented in Control Fibroblasts Treated with Depolymerizing Agents

**Figure 11:**
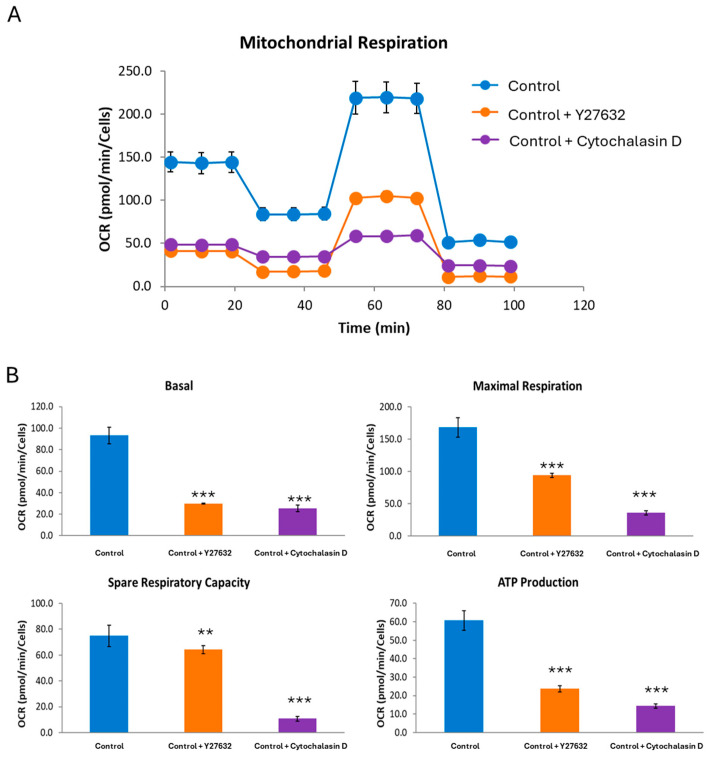
Bioenergetic analysis of control cells untreated and treated with Y27632 inhibitor or Cytochalasin D. (**A**) Respiratory profile of untreated and treated control cells (C1). (**B**) Basal, maximal, and spare respiratory capacity and ATP production were determined in untreated and treated control fibroblast using the Seahorse analyzer. Control fibroblasts were treated with 10 µM of Y27632 for 24 h or with 20 µM of Cytochalasin D for 3 h. ** *p* < 0.01, *** *p* < 0.001 between untreated and treated control cells.

As actin polymerization defects in NM patient-derived fibroblasts presented mitochondrial dysfunction [16], we next analyzed the effect of actin depolymerization by Y27632 inhibitor or Cytochalasin D on mitochondrial bioenergetics in control cells. Actin depolymerization in control fibroblasts induced a significant decrease in basal, spare, and maximal respiration as well as a lower mitochondrial ATP production (Figure 11A,B). 

### 2.8. Actin Depolymerizing Agents Induce Alterations in the Expression Levels of Iron Metabolism-Related Proteins

We also analyzed the effects of actin depolymerization by Y27632 or Cytochalasin D treatments on iron metabolism by studying the expression levels of several proteins involved in iron trafficking, storage, and regulation, including TFR1, DMT1, IRP1, Ferritin, Mitoferritin, Mitoferrin2, FXN, ISCU, Mt-ACP, and PANK2.

**Figure 12:**
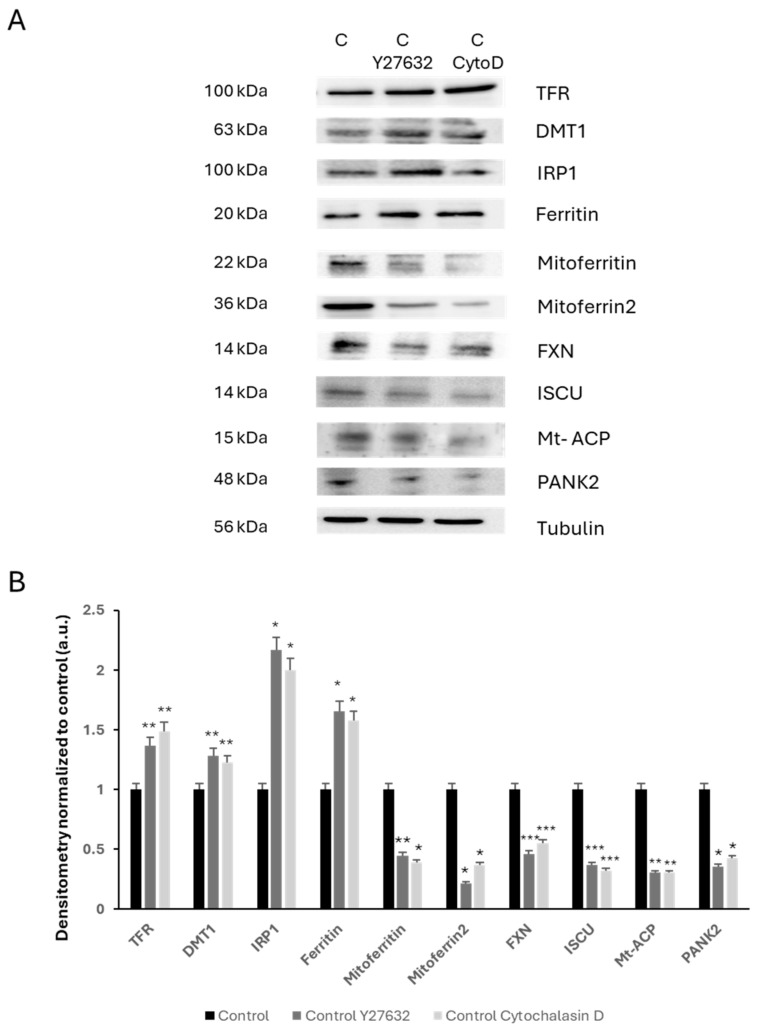
Expression levels of iron metabolism-related proteins in control fibroblasts treated with acting depolymerizing agents. (**A**) Immunoblotting analysis of cellular extracts from control cells (C1) treated with 10 µM of Y27632 for 24 h or 20 µM Cytochalasin D for 3 h. Protein extracts (50 μg) were separated on a SDS polyacrylamide gel and immunostained with antibodies against TFR, DMT1, IRP1, Ferritin, Mitoferritin, Mitoferrin2, FXN, ISCU, Mt-ACP, and PANK2. Tubulin was used as a loading control. (**B**) Densitometry of Western blotting. Data represent the mean ± SD of three separate experiments. * *p* < 0.05, ** *p* < 0.01, *** *p* < 0.001 between untreated and treated controls. A.U., arbitrary units.

As expected, actin depolymerization in controls cells treated with Y27632 or Cytochalasin D mimicked the dysregulated expression levels of iron metabolism-related proteins that were also observed in mutant nemaline cells (Figure 12A,B).

### 2.9. Actin Depolymerizing Agents Induce Iron and Lipofuscin Accumulation in Control Fibroblasts

To confirm the role of actin polymerization defects in iron overload, we analyzed the effects of Y27632 or Cytochalasin D treatments on iron accumulation in control cells by Prussian Blue staining.

**Figure 13:**
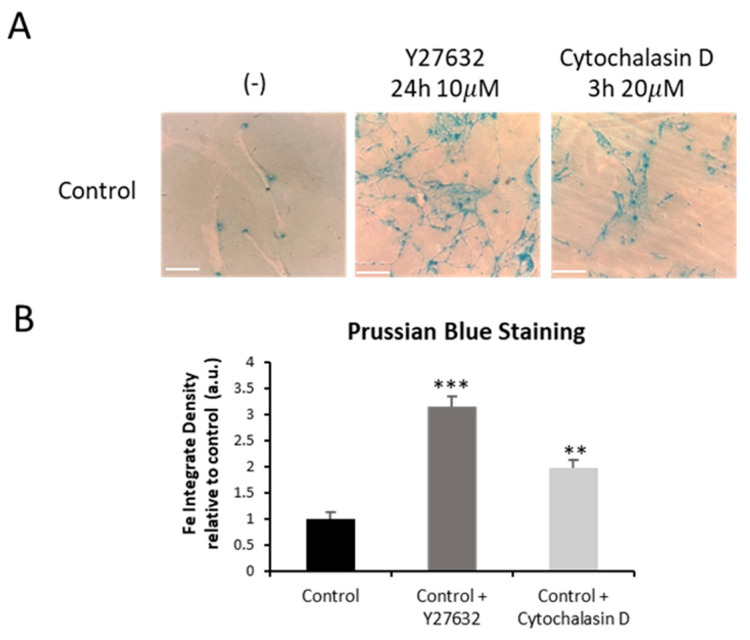
Iron accumulation in control cells treated with Y27632 or Cytochalasin D inhibitors. (**A**) Prussian Blue staining of control (C1) (−) (−) and control cells treated with 10 µM Y27632 for 24 h, or with 20 µM Cytochalasin D for 3 h. Images were made in brightfield by an Axio Vert A1 inverted optical microscope (Zeiss, Oberkochen, Germany) with a 40× objective and were analyzed using Fiji-ImageJ software (version 2.9.0/1.53t) (National Institute of Health, Bethesda, MD, USA). Scale bar = 20 µm. (**B**) Quantification of Prussian Blue staining images was performed by the Image J software (version 1.54f). Data represent the mean ± SD of three separate experiments. ** *p* < 0.01, *** *p* < 0.001 between untreated and treated control cells. A.U., arbitrary units.

Control fibroblasts treated with Y27632 or Cytochalasin D presented iron accumulation compared to untreated controls (Figure 13A,B).

**Figure 14:**
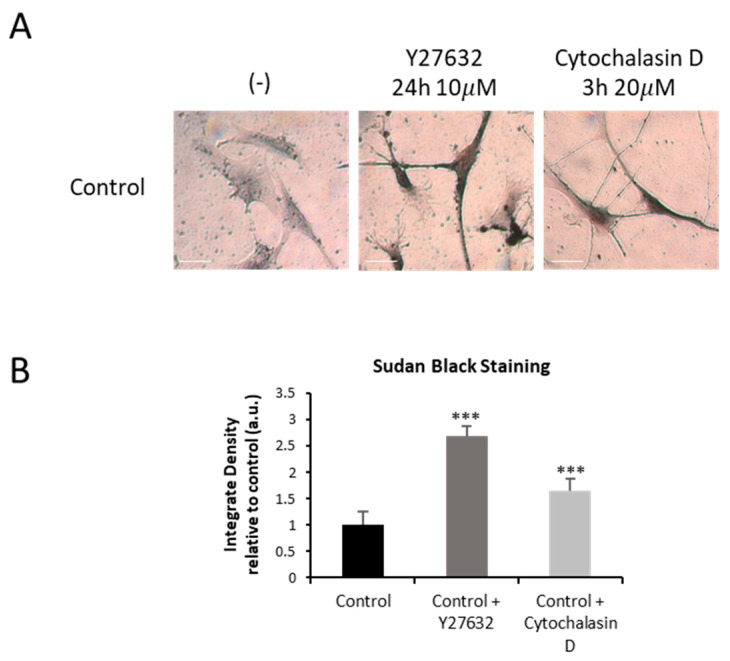
Lipofuscin accumulation in control cells treated with Y27632 or Cytochalasin D inhibitor. (**A**) Sudan Black staining of control cells (C1) treated with 10 µM Y27632 for 24 h, or with 20 µM Cytochalasin D for 3 h. Images were made in brightfield by an Axio Vert A1 inverted optical microscope (Zeiss, Oberkochen, Germany) with a 40× objective and were analyzed using Fiji-ImageJ software(version 2.9.0/1.53t) (National Institute of Health, Bethesda, MD, USA). Scale bar = 20 µm. (**B**) Quantification of Sudan Black staining images was performed by the Image J software (version 1.54f). Data represent the mean ± SD of three separate experiments. *** *p* < 0.001 between untreated and treated control cells. A.U., arbitrary units.

In addition, we analyzed the effect of Y27632 or Cytochalasin D inhibitors on the presence of lipofuscin accumulation in control fibroblasts by Sudan Black staining. As is shown in Figure 14, both actin depolymerizing agents induced an increased Sudan Black staining, suggesting lipofuscin accumulation.

### 2.10. Increased Lipid Peroxidation in Control Fibroblasts Treated with Y27632or Cytochalasin D

**Figure 15:**
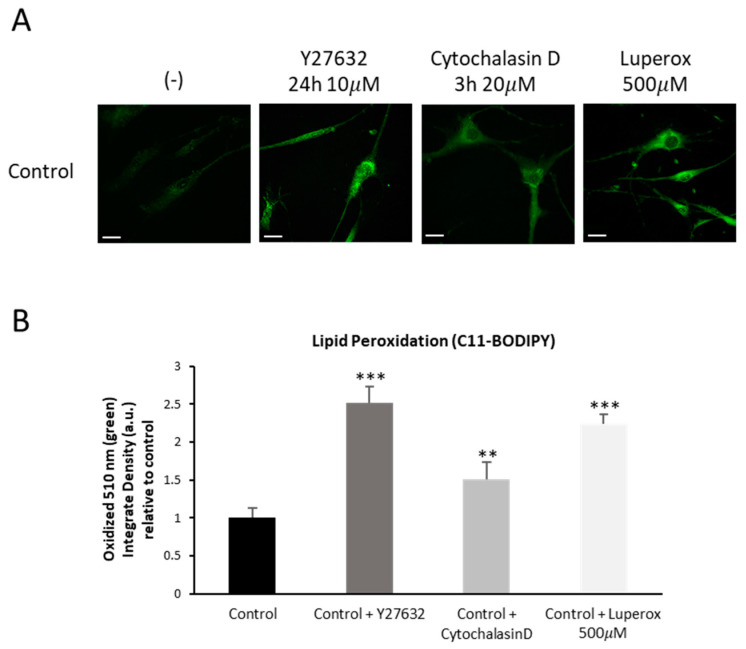
Cellular lipid peroxidation in control cells treated with Y27632 or Cytochalasin D inhibitors. (**A**) Control cells (C1) were treated with 10 µM Y27632 for 24 h, or with 20 µM Cytochalasin D for 3 h. The levels of cellular lipid peroxidation were measured using BODIPY^®^ staining as detailed in the Material and Methods. (**B**) BODIPY^®^ staining quantification was performed by using the Fiji software (version 2.9.0/1.53t). Control fibroblasts were treated with 500 µM Luperox (LUP, Tert-butyl hydroperoxide) for 15 min as positive control of lipid peroxidation. Scale bar = 20 µm. ** *p* < 0.01, *** *p* < 0.001 between treated and untreated control fibroblasts. Data represent the mean ± SD of four separate experiments. A.U., arbitrary units.

Next, we analyzed the effects of Y27632 or Cytochalasin D treatments on cellular lipid peroxidation. Comparing treated and untreated control fibroblasts, we observed that controls fibroblasts treated with both inhibitors showed elevated cellular lipid peroxidation (Figure 15A,B). Control fibroblasts were also treated with 500 µM Luperox (Tert-butyl hydroperoxide) as a positive control of lipid peroxidation.

## 3. Discussion

Nemaline myopathy (NM) is a rare subtype of congenital myopathy characterized by muscle weakness, hypotonia, and the presence of rods in the cytoplasm of muscle fibers, also known as nemaline bodies [7,8,10]. In this work, we analyzed iron accumulation and lipid peroxidation in NM cellular models using patient-derived fibroblasts harboring *ACTA1* and *NEB* pathogenic variants.

Recently, Piñero et al. described the interactions between actin polymerization deficiency and mitochondria function in *ACTA1* and *NEB* mutants [16]. Patient-derived fibroblasts stained by Rhodamine–Phalloidin showed defects and unstructured actin polymerization, as well as shorter length than control fibroblasts. The results obtained from the analysis of the expression levels of mitochondrial proteins such as NDUFA9 (complex I), NDUFS4 (complex I), mtND1 (complex I), SDHB (complex II), UQCRC2 (complex III), mtCO2 (complex IV), COX4 (complex IV), ATP5A (complex V), and VDAC1 showed that mutant fibroblasts exhibited reduced expression levels of these mitochondrial proteins.

In addition, mitochondrial morphology and dysfunction were analyzed using Mitotracker^TM^ staining, and mitochondrial respiratory function was evaluated by the Mitostress test assay in a XF24 extracellular flux analyzer [16]. The bioenergetic parameters analyzed showed significantly lower levels of mitochondrial respiration, basal and maximal respiration, reserve respiratory capacity, and mitochondrial ATP production. Moreover, mitochondrial dynamics were also found to be compromised in NM mutant fibroblasts. Two key proteins involved in mitochondrial fusion and fission, dynamin-related protein 1 (DRP1) and optic atrophy protein 1 (OPA1) [17,35], were also analyzed [16]. The results demonstrated elevated expression levels of DRP1 and reduced levels of OPA1, suggesting an imbalance between the processes of mitochondrial fusion and fission [16].

Particularly relevant for understanding NM pathophysiology is the relationship between mitochondrial dysfunction and iron metabolism. The mitochondrion upholds the synthesis of iron–sulfur clusters (ISCs) and heme, the most abundant iron-containing prosthetic groups in a large variety of proteins; therefore, a fraction of incoming iron must go through this organelle before reaching its final destination [30]. Apart of decreased ATP synthesis, mitochondrial dysfunction also results in decreased synthesis of ISCs and heme prosthetic groups and as a consequence may induce a dysregulation of iron metabolism [36]. In turn, the mitochondrial respiratory chain is the source of reactive oxygen species (ROS) derived from leaks in the electron transport chain. The co-existence of both iron and ROS in the secluded space of the mitochondrion makes this organelle particularly prone to hydroxyl radical-mediated damage. Mitochondrial dysfunction/iron overload has long been associated with several neurodegenerative diseases that include NBIA disorders, Alzheimer’s disease (AD), Huntington’s disease (HD), Parkinson’s disease (PD), amyotrophic lateral sclerosis (ALS), and Friedrich’s Ataxia (FA) [24,28,31,37,38,39].

In this work, for the first time, we described the association of mitochondrial dysfunction with iron accumulation in cellular models of NM (Figure 1 and Figure S1). Given that mutant cells showed increased autofluorescence spots which are positively stained with Sudan black, iron is presumably accumulated in the form of lipofuscin aggregates (Figure 2, Figure 3 and Figure S2). This hypothesis is also supported by the fact that deferiprone treatment eliminated both iron and autofluorescence/Sudan black spots. Lipofuscin granules have been previously reported in diaphragm and tibialis anterior muscles in NM mouse model carrying the human Met9Arg mutation of alpha-tropomyosin slow (Tpm3) [40] and muscle biopsies of a patient harboring a homozygous RYR1 c.8888T>C mutation [41]. Additionally, the TEM analysis shows the presence of elevated intracellular lipofuscin-like granules in NM fibroblasts harboring *ACTA1* and *NEB* mutations (Figure 4A,B).

As previously described, mitochondria play a crucial role in iron metabolism, since these organelles are a significant location for the use and accumulation of iron. Therefore, mitochondrial iron levels should be strictly regulated [27]. To analyze them, we examined the levels of mitochondrial ferrous iron (Fe^2+^) (Figure 5) in NM mutant and controls cells, indicating a dysfunction of iron metabolism and mitochondrial iron accumulation. In addition, TEM analysis showed the condensation and lateralization of damaged mitochondrial components which eventually formed lipofuscin granules (Figure 4C). This mechanism of mitochondrial lipofuscinogenesis may provide cellular protection from malfunctioning mitochondria [25].

Lipids are the primary structural elements of cellular membranes and subcellular organelles and play a crucial role in biological processes. Cell signaling, molecular transport, proliferation, energy storage, secretion, and survival are among their other crucial biological roles [24,42]. Furthermore, the lipid composition of the inner and outer mitochondrial membrane is also essential for the proper functioning of the electron transport chain, the stability of respiratory chain supercomplexes, and ATP production [43,44]. Excessive lipid oxidation may alter proteins and nucleic acids covalently and change the physicochemical characteristics of biological membranes. Lipid peroxidation and the oxidation of respiratory chain proteins are the two mechanisms that may cause mitochondrial dysfunction by an increase in ROS generation [45,46].

One of the main causes of cellular and tissue dysfunction that contributes to aging and most age-related and oxidative stress-related disorders is the accumulation of lipid peroxidation (LPO) products in human tissues [45,47]. It has been reported that lipid peroxidation in iron-rich organelles such as mitochondria may lead to iron accumulation in lipofuscin granules, which in turn may increase lipid peroxidation [24,31]. This vicious cycle in which lipid peroxidation and iron accumulation reinforce each other may participate in NM pathomechanisms.

Confirming this hypothesis, we demonstrated that iron overload in NM mutant cells was accompanied by increased lipid peroxidation. This interconnection between both pathological processes can aggravate the cellular damage, as recently described by our group [24]. Corroborating iron handling dysregulation, we found that NM patient-derived fibroblasts presented altered expression levels of proteins related to iron metabolism. Figure 6 shows the expression levels of proteins involved in iron homeostasis: TFR1, DMT1, IRP1, Ferritin, Mitoferritin, Mitoferrin2, FXN, ISCU, Mt- ACP, and PANK2. The elevated expression levels of TFR, DMT1, and Ferritin, proteins responsible for the iron transport and storage within cell, support the hypothesis of a dysregulation of iron metabolism in NM mutants. In addition, the expression levels of Frataxin (FXN) and ISCU, mitochondrial proteins involved in the biosynthesis of ISCs (iron–sulfur clusters), were downregulated compared with control fibroblast. ISCs are prosthetic groups that are attached to cytosolic and mitochondrial aconitases, as well as multiple subunits of mitochondrial respiratory complexes [48]. Consequently, a lack of proteins involved in ISC biogenesis may interfere with mitochondrial function, as previously described by our group [49]. Alterations in iron homeostasis are another consequence of this deficiency that may ultimately lead to mitochondrial iron excess. High iron levels in the mitochondria’s oxidative environment can then lead to a rise in ROS production and mitochondrial lipid peroxidation [50].

The paradoxical results of decreased LIP and iron accumulation have been also reported in PKAN cells by our research group. In additional works, *PANK2* silencing by siRNA in several human cell lines leads to a reduced proliferation rate, accompanied by a paradoxical iron deficiency and increased TfR1 expression levels [51]. Considering these observations, it has been proposed that the hypothesis that dysregulation of iron metabolism in mitochondria induces mitochondrial iron overload and cytosolic iron deficiency. The result is a vicious cycle characterized by increased iron uptake due to increased expression of Fe^2+^ transporters and subsequent accumulation in mitochondria and, finally, in lipofuscin granules [28,52]. This hypothesis could explain the mitochondrial dysfunction present in patients’ cells.

Interestingly, iron accumulation, increased lipid peroxidation, and mitochondrial dysfunction were reproduced in control cells treated with actin depolymerizing agents (Figure 10, Figure 11, Figure 12, Figure 13, Figure 14 and Figure 15), corroborating the essential role of actin filaments in mitochondrial function and consequently in iron metabolism and lipid peroxidation. There are several limitations in this study: (1) Only four patients have been included in this work. (2) Further research is needed to determine iron/lipofuscin accumulation and lipid peroxidation in animal models and muscle biopsies from patients.

## 4. Materials and Methods

### 4.1. Reagents

The following antibodies were purchased from Abcam (Cambridge, United Kingdom, UK): alpha-tubuline (ab7291), MTFRN2 Mitoferrin2 (ab80467), MTFRN1 Mitoferrin 1 (ab56134) (anti-FXN Frataxine (ab219414), and TFR Transferrin receptor (ab84036). The following antibodies were acquired from Santa Cruz (California (CA), United States (USA)): IRP1 iron-responsive element-binding protein (sc-166022), Ferritin (sc-74513), and PANK2 (sc-82288). ACP mit antibody was purchased from Invitrogen Thermo Fisher Scientific (Whaltham, Massachusetts (MA), United States (USA)). DMT1 antibody (Divalent Metal Transporter 1) ABS983 was acquired from EMD Millipore. ISCU antibody was acquired from GeneTex (California, (CA), United States (USA)). Perl’s Prussian Blue, Trypsin, dimethyl sulfoxide (DMSO), saponin, Tris base, 4′,6-diamidino-2-phenylindole (DAPI), and tetramethylethylenediamine (TEMED) were purchased from Sigma-Aldrich Chemical Co. (St. Louis, Missouri (MO), United States (USA)). Dulbecco’s modified Eagle’s medium (DMEM) with 4.5 g/L and 1 g/L glucose, L-glutamine, pyruvate, penicillin–streptomycin (10,000:10,000), and fetal bovine serum (FBS) were all sourced from Gibco. Mowiol 4-88 Mw was obtained from Sigma Chemical Co. (St. Louis, Missouri (MO), United States (USA)), while bovine serum albumin (BSA) was purchased from Santa Cruz Biotechnology (Paso Robles, California, (CA), United States (USA)). The Pierce™ BCA Protein Assay Kit was procured from Fisher Scientific (Whaltham, Massachusetts (MA), United States (USA)). Reagents to evaluate the mitochondrial activity were acquired from Santa Cruz Biotechnology (California, (CA), United States (USA)): oligomycin (sc-203342), carbonyl cyanide 4-(trifluoromethoxy) phenylhydrazone (FCCP) (sc-203578), and antimycin A (sc-202467A).

Various reagents were acquired from Bio-Rad Laboratories Inc. (Hercules, California (CA), United States (USA)), including Acrylamide 37.5:1 solution, Clarity™ Western ECL substrate, electrophoresis buffer (TGS), sodium dodecyl sulfate (SDS), Triton X-100, blot buffer (TG), Tween 20, and DC Protein Assay Reagents A, B, and S. Additional chemicals such as 2-propanol, ethanol, methanol, sodium chloride (NaCl), ammonium persulfate (APS), glacial acetic acid, potassium hydroxide (KOH), and potassium chloride (KCl) were supplied by Panreac (Barcelona, Spain). The Protease Inhibitor Cocktail was obtained from Roche (F. Hoffmann-La Roche Ltd., Basel, Switzerland). Y27632 (sc-3536) and paraformaldehyde (PFA; sc-25326B) were purchased from Santa Cruz Biotechnology (California (CA), United States (USA)).

### 4.2. Patients and Cell Cultures

Two controls lines of primary human skin fibroblasts were purchased from ATCC, and four lines of fibroblasts derived from patient skin biopsies from the Pediatric Department of Hospital Universitario Virgen del Rocío, Sevilla, Spain. Patient 1 (P1) presents a heterozygous pathogenic mutation c.133G>T (p.Val45Phe) in *ACTA1* that causes a missense variant. The second patient (P2) is also heterozygous, carrying changes in position c.760A>T (p.Asn254Tyr) in *ACTA1*. The third patient (P3) presents heterozygous pathogenic variants c.10321A>C (p.Thr3441Pro) and c.13669C>T (pArg4557*) in *NEB*. The fourth patient (P4) presents heterozygous pathogenic variants c.24407_24410dup (p.Leu8137Phefs*18) c.8425C>T (p.Arg2809*) in *NEB*.

Control values were represented as means ± SD of three control fibroblast cell lines. Fibroblasts were maintained in Dulbecco’s modified Eagle’s medium DMEM (Gibco™, ThermoFisher Scientific, Waltham, Massachusetts (MA), United States (USA)) supplemented with 10% FBS (Gibco™, ThermoFisher Scientific, Massachusetts (MA), United States (USA)), 100 mg/mL penicillin/streptomycin. Fibroblasts were cultured at 37 °C and 5% CO_2_. All the experiments were performed with fibroblasts’ cell cultures with a passage number < 10.

### 4.3. Determination of Iron and Lipofuscin Accumulation

Iron overload was determined by Prussian Blue staining and quantified in a microplate reader (Polar Star Omega, BMG Labtech, Ortenberg, Germany) and by brightfield microscopy. Images were taken by light and fluorescence Axio Vert A1 microscope (Zeiss, Oberkochen, Germany) with a 20× and 40× objective. A quantification analysis was performed by using the Image J software (version 1.54f). Moreover, iron content in cell extracts was also measured by inductively coupled plasma mass spectrometry (ICP-MS). Cell culture extracts were obtained by acid digestion with HNO_3_. Iron concentration was expressed as ng Fe^2+^/μg protein. Data were presented as means ± SD (standard deviation), *n* = 3 in all cases.

Lipofuscin granules were visualized by Sudan Black B (SBB) staining as previously described [53]. The quantification of SSB staining also was performed with the light and fluorescence Axio Vert A1 microscope (Zeiss, Oberkochen, Germany) with a 20× and 40× objective. The cell’s autofluorescence was measured by fluorescence microscopy (excitation 366 nm; emission 420–600 nm). Confocal laser scanning microscopy (Nikon A1R, Shinagawa, Tokyo, Japan) was used for obtaining the emission spectra of lipofuscin granules as previously described [28]. The emission spectra were recorded in 20 lipofuscin granules in 20 cells. All images were analyzed by Fiji-ImageJ software (version 2.9.0/1.53t).

In both determinations, fibroblasts were treated with 100 μM deferiprone (Def) [28], an iron chelating drug which were used as a negative control to corroborate the specificity of the conducted staining’s and techniques.

### 4.4. TEM Analysis

Transmission electron microscopy was performed following the protocol previously described by our group [28,54]. Control and patients’ cells were seeded on 8-well Permanox chamber slides (Nunc, Thermo Scientific) and were subsequently fixed in tempered 3.5% glutaraldehyde in 0.1 M phosphate buffer (PB) for 5 min at 37 °C and 55 min for 4 °C. Cells were postfixed in 2% OsO4 for 1 h at room temperature, rinsed, dehydrated, and embedded in Durcupan resin (Fluka, Sigma-Aldrich). Semithin sections (1.5 µm) were cut with a diamond knife and stained lightly with 1% toluidine blue. Later, ultra-thin (70 nm) sections of the cells were cut with a diamond knife, stained with lead citrate (Reynolds solution), and examined under a transmission electron microscope (FEI Tecnai G2 Spirit BioTwin) with a Xarosa (20 Megapixel resolution) digital camera using Radius image acquisition software (version 2.1, EMSIS GmbH, Münster, Germany).

### 4.5. Measurement of Mitochondrial Iron Accumulation (Mito-FerroGreen)

Mitochondrial iron levels were measured by Mito-FerroGreen staining, a novel fluorescent probe for the detection of ferrous ion (Fe^2+^) in mitochondria where Fe-S clusters and heme proteins are synthesized and enables live cell fluorescent imaging of intracellular Fe^2+^. Mito-FerroGreen was obtained from the Dojindo Laboratory (Kumamoto, Japan). Fibroblasts were washed three times with HBSS supplemented with Ca^2+^ and Mg^2+^ and incubated for 30 min at 37 °C with 5 µM Mito-FerroGreen and 100 nM MitoTracker™ Deep Red FM for 45 min at 37 °C. Then, cells were washed three times with HBSS supplemented with Ca^2+^ and Mg^2+^. Images were taken using a DeltaVision system (version softWoRx 7.0; Applied Precision; Issaquah, Washington (WA), United States (USA)) with an Olympus IX-71 fluorescence microscope (Olympus Corporation, Shinjuku, Tokyo, Japan) with a 40× oil objective and analyzed by Fiji-ImageJ software (version 2.9.0/1.53t). To corroborate the specific Mito-FerroGreen presence in the mitochondria, cells were co-stained with MitoTracker™ Deep Red FM, and the Pearson correlation coefficient was calculated using the JaCoP plugin from Fiji-ImageJ software (version 2.9.0/1.53t). A positive correlation was considered when Pearson coefficient >0.75. Mitochondrial iron levels were determined by quantification of the Mito-FerroGreen fluorescence intensity from 30 cells. Data were presented as means ± SD (standard deviation), *n* = 3 in all cases.

### 4.6. Inmunoblotting

Western blotting was performed using standard methods. After protein transfer, the membrane was incubated with various primary antibodies diluted 1:1000 and then with the corresponding secondary antibody coupled to horseradish peroxidase at a 1:10,000 dilution. Specific protein complexes were identified using the Immun-Star HRP substrate kit (Biorad Laboratories Inc., Hercules, California (CA), United States (USA)).

### 4.7. Measurement of Labile Iron Pool

Labile iron pool (LIP) determination was carried out as a slightly modified version of what has been previously described [33]. Briefly, cells were seeded in 96-well plates. Control and patients’ fibroblasts were incubated in the medium supplemented with 1 mg/mL BSA and 0.25 μM calcein-AM for 30 min at 37 °C. After two washes with Hank’s Balanced Salt Solution (HBSS), cells were maintained in HBSS supplemented with 5 mM glucose, 20 mM HEPES, and 15 mM NaCl for 10 min at 37 °C. Basal fluorescence was measured using a Polar Star Omega Microplate Reader at 485 nm (excitation) and 535 nm (emission). Cells were then supplemented with Salicyladehyde Isonicotinoyl Hydrazone (0.1 mM), a specific iron chelator, for 15 min. Fluorescence was monitored during incubation with the chelator, and when a plateau was reached, that value was the LIP value. The final determination of the LIP level is carried out using the ratio LIP value/basal measurement, and the results were normalized to the protein content (mg of protein). The control cells were previously treated with 100 μM deferiprone as a negative control. Data were presented as means ± SD (standard deviation), *n* = 3 in all cases.

### 4.8. Measurement of Membrane Lipid Peroxidation

Lipid peroxidation was determined using 4,4-difluoro-5-(4-phenyl-1,3-butadienyl)-4-bora-3a,4a-diaza-s-indacene-3-undecanoic acid (BODIPY^®^ 581/591 C11) (D3861, ThermoFisher Scientific), a lipophilic fluorescent dye [25,55]. Cells were incubated with 5 μM BODIPY^®^ 581/591 C11 for 30 min at 37 °C. Luperox^®^ TBH70X (458139, Sigma-Aldrich) at 500 μM for 15 min was used as positive control of lipid peroxidation. Lipid peroxidation in fibroblasts was evaluated by an Axio Vert A1 fluorescence microscope with a 40× objective. All images were analyzed by Fiji-ImageJ software (version 2.9.0/1.53t). Data were presented as means ± SD (standard deviation), *n* = 3 in all cases.

### 4.9. Measurement of Mitochondrial Lipid Peroxidation (MitoPeDPP)

Mitochondrial lipid peroxidation was evaluated using a [3-(4-phenoxyphenylpyrenylphosphino) propyl] triphenylphosphonium iodide fluorescent probe (MitoPeDPP^®^) developed by Shioji K., et al. [56]. Localization of the MitoPeDPP signal in mitochondria was addressed by a colocalization analysis with MitoTracker™ Deep Red FM, an in vivo mitochondrial dye. Control and patients’ cells were treated with 300 nM MitoPeDPP^®^ for 15 min at 37 °C and 100 nM MitoTracker™ Deep Red FM for 45 min at 37 °C. Luperox^®^ TBH70X (458139, Sigma-Aldrich) at 500 μM for 15 min was used as positive control of lipid peroxidation. Images were taken by DeltaVision (version softWoRx 7.0; Applied Precision; Issaquah, Washington (WA), United states (USA)) system with an Olympus IX-71 fluorescence microscope(Olympus Corporation, Shinjuku, Tokyo, Japan) with a 40× oil objective and analyzed by Fiji-ImageJ software (version 2.9.0/1.53t). Data were presented as means ± SD (standard deviation), *n* = 3 in all cases.

### 4.10. Analysis of Mitochondrial Network and Cytoskeletal F-Actin

In order to evaluate the effect of actin inhibitors Y27632 and Cytochalasin D on the state of actin filaments and the mitochondrial network, control and patients’ fibroblasts were stained with 1 μg/mL Rhodamine–Phalloidin for 30 min and 100 nM MitoTracker™ Deep Red FM for 45 min at 37 °C. Evaluation of mitochondrial network and cytoskeletal F-actin was conducted following our previously described protocols [16]. All images were taken by a DeltaVision (version softWoRx 7.0; Applied Precision; Issaquah, Washington (WA), United states (USA)) system with an Olympus IX-71 fluorescence microscope with a 40× oil objective and analyzed by Fiji-ImageJ software (version 2.9.0/1.53t).

### 4.11. Bioenergetics and Oxidative Stress Analysis

An XF24 extracellular flux analyzer (Seahorse Bioscience, Billerica, Massachusetts (MA), United States (USA)) was used to perform a Mitostress test experiment to assess the mitochondrial respiratory performance of untreated and treated control with inhibitors Y27632 and Cytochalasin D. In XF24 cell culture plates, cells were cultivated at a density of 15,000 cells per well with 150 μL of growth media (DMEM supplemented with 20% FBS) at 37 °C and 5% CO_2_. Only 50 μL of media remained after the growth medium from each well was removed after the incubation period of 24 h. Following two rounds of cell washing with 1 mL of pre-warmed assay medium (XF base media supplemented with 10 mM glucose, 1 mM glutamine, and 1 mM sodium pyruvate; pH 7.4), 450 μL of assay medium (500 μL total) was added to each well. Fibroblasts pre-equilibrate with the test media by being cultured for an hour at 37 °C without CO_2_. Four different compounds that impact bioenergetics were injected sequentially to test mitochondrial activity. The final doses of these four compounds were administered as follows: 1 μM oligomycin, 2 μM FCCP (carbonyl cyanide-4-trifluoromethoxy-phenylhydrazone), and 2.5 μM antimycin A/rotenone.

To determine the ideal cell seeding density and the ideal dose of each inhibitor and uncoupler, preliminary tests were performed. A minimum of five wells have been used for each treatment in each experiment. Important mitochondrial characteristics, including ATP synthesis, spare respiratory capacity, and basal and maximal respiration, may be estimated with this test. The XF24 analyzer’s results were standardized according to the 15,000 cells that were planted. The BioTek^TM^ Cytation^TM^ 1 Cell Imaging Multi-Mode Reader was used to count the cells in each well both before and after the experiment to evaluate if the number of cells remained constant.

### 4.12. Statistics

Statistical analysis was conducted in accordance with our research group’s previous description [16]. We used non-parametric statistics, where there were few events (*n <* 30), that do not have any distributional assumption, given the low reliability of normality testing for small sample sizes used in this work. In these cases, multiple groups were compared using a Kruskal–Wallis test. We used parametric tests when the number of events was greater (*n* > 30). In these instances, a one-way ANOVA was used to compare multiple groups. Statistical analyses were conducted using the GraphPad Prism 9.0 (GraphPad Software, San Diego, CA, USA). All results were presented as the mean ± SD values or as an example from 3 independent experiments, and *p*-values of less than 0.05 were considered significant.

## 5. Conclusions

In conclusion, our findings demonstrate that fibroblasts derived from patients with NM are useful cellular models to study disease pathophysiology. Furthermore, we confirm the close relationship between the actin cytoskeleton, mitochondria function, iron metabolism, and lipid peroxidation. Patient-derived cellular models may complement *ACTA1* and *NEB* mouse and zebrafish models to understand disease pathomechanisms and enable the evaluation of genomic or pharmacological therapies.

## Data Availability

Data supporting the findings of this study are not openly available due to reasons of sensitivity and to protect the privacy of individuals; however, data are available from the corresponding author upon reasonable request. Data are located in controlled access data storage at Pablo de Olavide University (https://jazmin.upo.es/bscw/bscw.cgi).

## Supplementary Materials

The following supporting information can be downloaded at: https://www.mdpi.com/article/10.3390/ijms26041434/s1.

## References

[1] North K.N., Wang C.H., Clarke N., Jungbluth H., Vainzof M., Dowling J.J., Amburgey K., Quijano-Roy S., Beggs A.H., Sewry C. (2014). Approach to the diagnosis of congenital myopathies. Neuromuscul. Disord..

[2] Ogasawara M., Nishino I. (2023). A review of major causative genes in congenital myopathies. J. Hum. Genet..

[3] Cassandrini D., Trovato R., Rubegni A., Lenzi S., Fiorillo C., Baldacci J., Minetti C., Astrea G., Bruno C., Santorelli F.M. (2017). Congenital myopathies: Clinical phenotypes and new diagnostic tools. Ital. J. Pediatr..

[4] Christophers B., Lopez M.A., Gupta V.A., Vogel H., Baylies M. (2022). Pediatric Nemaline Myopathy: A Systematic Review Using Individual Patient Data. J. Child. Neurol..

[5] Conen P.E., Murphy E.G., Donohue W.L. (1963). Light and Electron Microscopic Studies of “Myogranules” in a Child with Hypotonia and Muscle Weakness. Can. Med. Assoc. J..

[6] Shy G.M., Engel W.K., Somers J.E., Wanko T. (1963). Nemaline Myopathy. A New Congenital Myopathy. Brain.

[7] Laitila J., Wallgren-Pettersson C. (2021). Recent advances in nemaline myopathy. Neuromuscul. Disord..

[8] Malfatti E., Romero N.B. (2016). Nemaline myopathies: State of the art. Rev. Neurol..

[9] Yin X., Pu C.Q., Wang Q., Liu J.X., Mao Y.L. (2014). Clinical and pathological features of patients with nemaline myopathy. Mol. Med. Rep..

[10] de Winter J.M., Ottenheijm C.A.C. (2017). Sarcomere Dysfunction in Nemaline Myopathy. J. Neuromuscul. Dis..

[11] Fabian L., Karimi E., Farman G.P., Gohlke J., Ottenheijm C.A.C., Granzier H.L., Dowling J.J. (2024). Comprehensive phenotypic characterization of an allelic series of zebrafish models of NEB-related nemaline myopathy. Hum. Mol. Genet..

[12] Yamaguchi M., Robson R.M., Stromer M.H., Dahl D.S., Oda T. (1982). Nemaline myopathy rod bodies. Structure and composition. J. Neurol. Sci..

[13] Lamont P.J., Thorburn D.R., Fabian V., Vajsar J., Hawkins C., Saada Reisch A., Durling H., Laing N.G., Nevo Y. (2004). Nemaline rods and complex I deficiency in three infants with hypotonia, motor delay and failure to thrive. Neuropediatrics.

[14] Pula S., Urankar K., Norman A., Pierre G., Langton-Hewer S., Selby V., Mason F., Vijayakumar K., McFarland R., Taylor R.W. (2020). A novel de novo ACTA1 variant in a patient with nemaline myopathy and mitochondrial Complex I deficiency. Neuromuscul. Disord..

[15] Illescas M., Peñas A., Arenas J., Martín M.A., Ugalde C. (2021). Regulation of Mitochondrial Function by the Actin Cytoskeleton. Front. Cell Dev. Biol..

[16] Pinero-Perez R., Lopez-Cabrera A., Alvarez-Cordoba M., Cilleros-Holgado P., Talaveron-Rey M., Suarez-Carrillo A., Munuera-Cabeza M., Gomez-Fernandez D., Reche-Lopez D., Romero-Gonzalez A. (2023). Actin Polymerization Defects Induce Mitochondrial Dysfunction in Cellular Models of Nemaline Myopathies. Antioxidants.

[17] Fenton A.R., Jongens T.A., Holzbaur E.L.F. (2021). Mitochondrial dynamics: Shaping and remodeling an organelle network. Curr. Opin. Cell Biol..

[18] Fernie A.R., Zhang Y., Sampathkumar A. (2020). Cytoskeleton Architecture Regulates Glycolysis Coupling Cellular Metabolism to Mechanical Cues. Trends Biochem. Sci..

[19] Kast D.J., Dominguez R. (2017). The Cytoskeleton-Autophagy Connection. Curr. Biol..

[20] Moore A.S., Wong Y.C., Simpson C.L., Holzbaur E.L. (2016). Dynamic actin cycling through mitochondrial subpopulations locally regulates the fission-fusion balance within mitochondrial networks. Nat. Commun..

[21] Tilokani L., Nagashima S., Paupe V., Prudent J. (2018). Mitochondrial dynamics: Overview of molecular mechanisms. Essays Biochem..

[22] Ni S., Yuan Y., Kuang Y., Li X. (2022). Iron Metabolism and Immune Regulation. Front. Immunol..

[23] Vogt A.S., Arsiwala T., Mohsen M., Vogel M., Manolova V., Bachmann M.F. (2021). On Iron Metabolism and Its Regulation. Int. J. Mol. Sci..

[24] Villalon-Garcia I., Povea-Cabello S., Alvarez-Cordoba M., Talaveron-Rey M., Suarez-Rivero J.M., Suarez-Carrillo A., Munuera-Cabeza M., Reche-Lopez D., Cilleros-Holgado P., Pinero-Perez R. (2023). Vicious cycle of lipid peroxidation and iron accumulation in neurodegeneration. Neural Regen. Res..

[25] Suarez-Carrillo A., Alvarez-Cordoba M., Romero-Gonzalez A., Talaveron-Rey M., Povea-Cabello S., Cilleros-Holgado P., Pinero-Perez R., Reche-Lopez D., Gomez-Fernandez D., Romero-Dominguez J.M. (2023). Antioxidants Prevent Iron Accumulation and Lipid Peroxidation, but Do Not Correct Autophagy Dysfunction or Mitochondrial Bioenergetics in Cellular Models of BPAN. Int. J. Mol. Sci..

[26] Nakamura T., Naguro I., Ichijo H. (2019). Iron homeostasis and iron-regulated ROS in cell death, senescence and human diseases. Biochim. Biophys. Acta Gen. Subj..

[27] Paul B.T., Manz D.H., Torti F.M., Torti S.V. (2017). Mitochondria and Iron: Current questions. Expert Rev. Hematol..

[28] Alvarez-Cordoba M., Fernandez Khoury A., Villanueva-Paz M., Gomez-Navarro C., Villalon-Garcia I., Suarez-Rivero J.M., Povea-Cabello S., de la Mata M., Cotan D., Talaveron-Rey M. (2019). Pantothenate Rescues Iron Accumulation in Pantothenate Kinase-Associated Neurodegeneration Depending on the Type of Mutation. Mol. Neurobiol..

[29] Gomez-Fernandez D., Romero-Gonzalez A., Suarez-Rivero J.M., Cilleros-Holgado P., Alvarez-Cordoba M., Pinero-Perez R., Romero-Dominguez J.M., Reche-Lopez D., Lopez-Cabrera A., Ibanez-Mico S. (2024). A Multi-Target Pharmacological Correction of a Lipoyltransferase LIPT1 Gene Mutation in Patient-Derived Cellular Models. Antioxidants.

[30] Urrutia P.J., Mena N.P., Núñez M.T. (2014). The interplay between iron accumulation, mitochondrial dysfunction, and inflammation during the execution step of neurodegenerative disorders. Front. Pharmacol..

[31] Villalon-Garcia I., Alvarez-Cordoba M., Povea-Cabello S., Talaveron-Rey M., Villanueva-Paz M., Luzon-Hidalgo R., Suarez-Rivero J.M., Suarez-Carrillo A., Munuera-Cabeza M., Salas J.J. (2022). Vitamin E prevents lipid peroxidation and iron accumulation in PLA2G6-Associated Neurodegeneration. Neurobiol. Dis..

[32] Bindewald-Wittich A., Han M., Schmitz-Valckenberg S., Snyder S.R., Giese G., Bille J.F., Holz F.G. (2006). Two-photon-excited fluorescence imaging of human RPE cells with a femtosecond Ti:Sapphire laser. Investig. Ophthalmol. Vis. Sci..

[33] Campanella A., Privitera D., Guaraldo M., Rovelli E., Barzaghi C., Garavaglia B., Santambrogio P., Cozzi A., Levi S. (2012). Skin fibroblasts from pantothenate kinase-associated neurodegeneration patients show altered cellular oxidative status and have defective iron-handling properties. Hum. Mol. Genet..

[34] Orellana D.I., Santambrogio P., Rubio A., Yekhlef L., Cancellieri C., Dusi S., Giannelli S.G., Venco P., Mazzara P.G., Cozzi A. (2016). Coenzyme A corrects pathological defects in human neurons of PANK2-associated neurodegeneration. EMBO Mol. Med..

[35] Noone J., O’Gorman D.J., Kenny H.C. (2022). OPA1 regulation of mitochondrial dynamics in skeletal and cardiac muscle. Trends Endocrinol. Metab..

[36] Munoz Y., Carrasco C.M., Campos J.D., Aguirre P., Nunez M.T. (2016). Parkinson’s Disease: The Mitochondria-Iron Link. Park. Dis..

[37] Alvarez-Cordoba M., Talaveron-Rey M., Villalon-Garcia I., Povea-Cabello S., Suarez-Rivero J.M., Suarez-Carrillo A., Munuera-Cabeza M., Salas J.J., Sanchez-Alcazar J.A. (2021). Down regulation of the expression of mitochondrial phosphopantetheinyl-proteins in pantothenate kinase-associated neurodegeneration: Pathophysiological consequences and therapeutic perspectives. Orphanet J. Rare Dis..

[38] Grubman A., White A.R., Liddell J.R. (2014). Mitochondrial metals as a potential therapeutic target in neurodegeneration. Br. J. Pharmacol..

[39] Talaveron-Rey M., Alvarez-Cordoba M., Villalon-Garcia I., Povea-Cabello S., Suarez-Rivero J.M., Gomez-Fernandez D., Romero-Gonzalez A., Suarez-Carrillo A., Munuera-Cabeza M., Cilleros-Holgado P. (2023). Alpha-lipoic acid supplementation corrects pathological alterations in cellular models of pantothenate kinase-associated neurodegeneration with residual PANK2 expression levels. Orphanet J. Rare Dis..

[40] Sanoudou D., Corbett M.A., Han M., Ghoddusi M., Nguyen M.A., Vlahovich N., Hardeman E.C., Beggs A.H. (2006). Skeletal muscle repair in a mouse model of nemaline myopathy. Hum. Mol. Genet..

[41] Bohm J., Vasli N., Malfatti E., Le Gras S., Feger C., Jost B., Monnier N., Brocard J., Karasoy H., Gerard M. (2013). An integrated diagnosis strategy for congenital myopathies. PLoS ONE.

[42] Santos A.L., Preta G. (2018). Lipids in the cell: Organisation regulates function. Cell Mol. Life Sci..

[43] Martensson C.U., Doan K.N., Becker T. (2017). Effects of lipids on mitochondrial functions. Biochim. Biophys. Acta Mol. Cell Biol. Lipids.

[44] Mejia E.M., Hatch G.M. (2016). Mitochondrial phospholipids: Role in mitochondrial function. J. Bioenerg. Biomembr..

[45] Ademowo O.S., Dias H.K.I., Burton D.G.A., Griffiths H.R. (2017). Lipid (per) oxidation in mitochondria: An emerging target in the ageing process?. Biogerontology.

[46] Paradies G., Paradies V., Ruggiero F.M., Petrosillo G. (2014). Oxidative stress, cardiolipin and mitochondrial dysfunction in nonalcoholic fatty liver disease. World J. Gastroenterol..

[47] Negre-Salvayre A., Auge N., Ayala V., Basaga H., Boada J., Brenke R., Chapple S., Cohen G., Feher J., Grune T. (2010). Pathological aspects of lipid peroxidation. Free. Radic. Res..

[48] Lill R., Srinivasan V., Muhlenhoff U. (2014). The role of mitochondria in cytosolic-nuclear iron-sulfur protein biogenesis and in cellular iron regulation. Curr. Opin. Microbiol..

[49] Maio N., Rouault T.A. (2022). Mammalian iron sulfur cluster biogenesis and human diseases. IUBMB Life.

[50] Lu C., Cortopassi G. (2007). Frataxin knockdown causes loss of cytoplasmic iron-sulfur cluster functions, redox alterations and induction of heme transcripts. Arch. Biochem. Biophys..

[51] Poli M., Derosas M., Luscieti S., Cavadini P., Campanella A., Verardi R., Finazzi D., Arosio P. (2010). Pantothenate kinase-2 (Pank2) silencing causes cell growth reduction, cell-specific ferroportin upregulation and iron deregulation. Neurobiol. Dis..

[52] Huang M.L., Lane D.J., Richardson D.R. (2011). Mitochondrial mayhem: The mitochondrion as a modulator of iron metabolism and its role in disease. Antioxid. Redox Signal..

[53] Georgakopoulou E.A., Tsimaratou K., Evangelou K., Fernandez Marcos P.J., Zoumpourlis V., Trougakos I.P., Kletsas D., Bartek J., Serrano M., Gorgoulis V.G. (2013). Specific lipofuscin staining as a novel biomarker to detect replicative and stress-induced senescence. A method applicable in cryo-preserved and archival tissues. Aging.

[54] Rodriguez-Hernandez A., Cordero M.D., Salviati L., Artuch R., Pineda M., Briones P., Gomez Izquierdo L., Cotan D., Navas P., Sanchez-Alcazar J.A. (2009). Coenzyme Q deficiency triggers mitochondria degradation by mitophagy. Autophagy.

[55] Pap E.H., Drummen G.P., Winter V.J., Kooij T.W., Rijken P., Wirtz K.W., Op den Kamp J.A., Hage W.J., Post J.A. (1999). Ratio-fluorescence microscopy of lipid oxidation in living cells using C11-BODIPY(581/591). FEBS Lett..

[56] Shioji K., Oyama Y., Okuma K., Nakagawa H. (2010). Synthesis and properties of fluorescence probe for detection of peroxides in mitochondria. Bioorganic Med. Chem. Lett..

