# Supplementary material for: Iron Accumulation and Lipid Peroxidation in Cellular Models of Nemaline Myopathies
Source: Int J Mol Sci. 2025 Feb 8;26(4):1434. doi: 10.3390/ijms26041434 (PMC11855326; doi:10.3390/ijms26041434)
Supplement: Supplementary file 1 [file ijms-26-01434-s001.zip › ijms-3359857-supplementary.pdf]

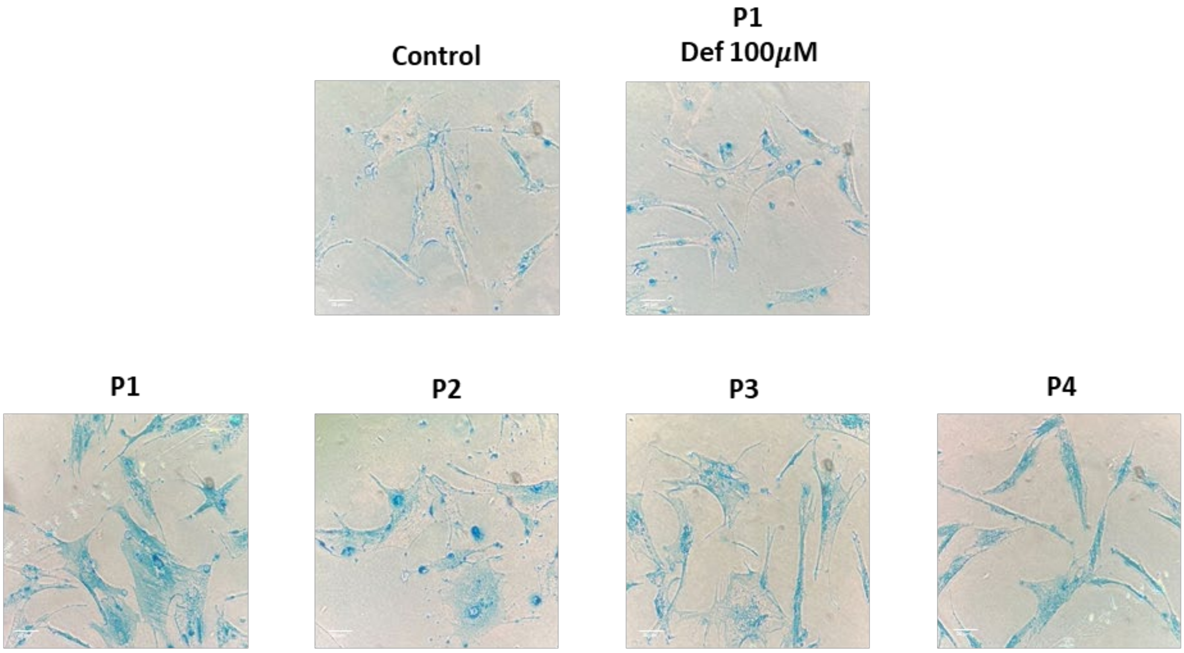

Supplementary figure 1. Iron accumulation in NM patient-derived fibroblasts. Prussian Blue staining of controls cells and NM fibroblasts was performed as described in Materials and Methods. P1 fibroblasts were exposed to 100 µM deferiprone (Def), an iron chelating agent, for 24 hours, as a negative control. Axio Vert A1 inverted optical microscope (Zeiss, Oberkochen, Germany) with a 20x objective was used to make the images. Scale Bar = 20µm.

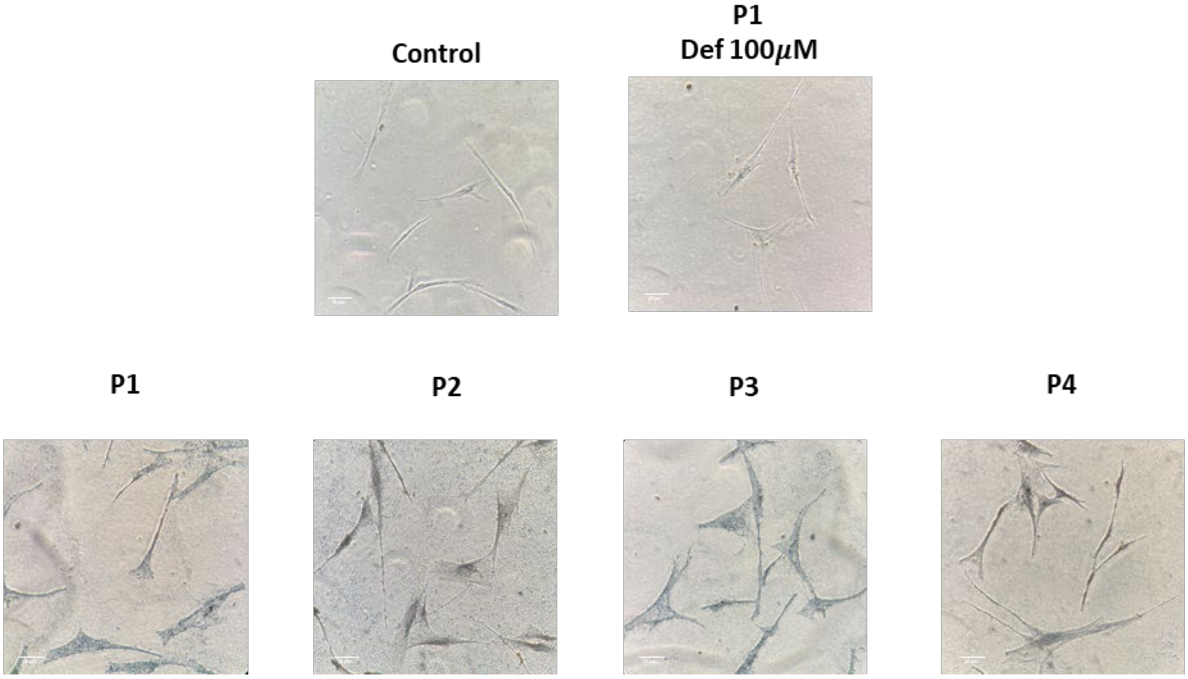

Supplementary figure 2. Lipofuscin accumulation in NM patient-derived fibroblasts. Sudan Black staining of controls cells and NM fibroblasts was performed as described in Materials and Methods. P1 fibroblasts were exposed to 100 μM deferiprone (Def), an iron chelating agent, for 24 hours, as a negative control. Axio Vert A1 inverted optical microscope (Zeiss, Oberkochen, Germany) with a 20x objective was used to make the images. Scale Bar = 20μm.
